# A primary isolated heart model of Takotsubo syndrome for mechanistic investigation and preclinical therapeutic testing

**DOI:** 10.1093/ehjopen/oeag141

**Published:** 2026-09-15

**Authors:** Amirali Nejat, Ermir Zulfaj, Yalda Kakaei, Mana Kalani, Tetiana Pylova, Maryna Krasnikova, Ahmed Elmahdi, Sandeep Jha, Björn Redfors, Elmir Omerovic

**Affiliations:** Department of Molecular and Clinical Medicine, Institute of Medicine, Sahlgrenska Academy at Gothenburg University, Bruna stråket 16, 413 45 Gothenburg, Sweden; Department of Molecular and Clinical Medicine, Institute of Medicine, Sahlgrenska Academy at Gothenburg University, Bruna stråket 16, 413 45 Gothenburg, Sweden; Department of Molecular and Clinical Medicine, Institute of Medicine, Sahlgrenska Academy at Gothenburg University, Bruna stråket 16, 413 45 Gothenburg, Sweden; Department of Molecular and Clinical Medicine, Institute of Medicine, Sahlgrenska Academy at Gothenburg University, Bruna stråket 16, 413 45 Gothenburg, Sweden; Department of Molecular and Clinical Medicine, Institute of Medicine, Sahlgrenska Academy at Gothenburg University, Bruna stråket 16, 413 45 Gothenburg, Sweden; Department of Molecular and Clinical Medicine, Institute of Medicine, Sahlgrenska Academy at Gothenburg University, Bruna stråket 16, 413 45 Gothenburg, Sweden; Department of Molecular and Clinical Medicine, Institute of Medicine, Sahlgrenska Academy at Gothenburg University, Bruna stråket 16, 413 45 Gothenburg, Sweden; Department of Molecular and Clinical Medicine, Institute of Medicine, Sahlgrenska Academy at Gothenburg University, Bruna stråket 16, 413 45 Gothenburg, Sweden; Department of Cardiology, Sahlgrenska University Hospital, Gothenburg, Sweden, 41346 Gothenburg, Sweden; Department of Molecular and Clinical Medicine, Institute of Medicine, Sahlgrenska Academy at Gothenburg University, Bruna stråket 16, 413 45 Gothenburg, Sweden; Department of Cardiology, Sahlgrenska University Hospital, Gothenburg, Sweden, 41346 Gothenburg, Sweden; Department of Molecular and Clinical Medicine, Institute of Medicine, Sahlgrenska Academy at Gothenburg University, Bruna stråket 16, 413 45 Gothenburg, Sweden; Department of Cardiology, Sahlgrenska University Hospital, Gothenburg, Sweden, 41346 Gothenburg, Sweden

**Keywords:** Takotsubo syndrome, Catecholamine, Echocardiography, Langendorff perfusion, Preclinical therapeutic testing

## Abstract

**Aims:**

Current experimental models of Takotsubo syndrome (TTS) do not readily distinguish intrinsic myocardial responses from systemic neurohumoral influences. We sought to establish and comprehensively characterize a primary isolated heart TTS model, in which the complete TTS-like phenotype is induced entirely within a Langendorff-perfused rat heart, enabling mechanistic investigation and preclinical therapeutic testing under controlled conditions.

**Methods and results:**

Male Sprague-Dawley rat hearts were perfused, and TTS was induced by controlled intra-aortic isoprenaline infusion, while control hearts received vehicle alone. Haemodynamic function and coronary flow were monitored continuously for 6 h. The primary endpoint was the group-by-time interaction for left ventricular developed pressure, analysed using generalized additive mixed models. Prespecified exploratory analyses included principal component analysis, Gaussian mixture modelling, and machine-learning-based feature analysis. Echocardiography, cardiac troponin release, and representative histology were used for multimodal phenotypic validation. Primary isolated TTS hearts developed progressive systolic and diastolic dysfunction characterized by reductions in left ventricular developed pressure, rate-pressure product, and coronary flow together with prolongation of surrogate isovolumic relaxation constant (TAU) (primary endpoint: *P* = 0.001; *q* = 0.002). Temporal modelling identified three reproducible physiological phases: hyperactivation, transition, and functional nadir. Echocardiography demonstrated characteristic apical ballooning, reduced ejection fraction, impaired global longitudinal strain, and increased left ventricular akinesia index. Progressive cardiac troponin release and representative histological alterations provided supportive biochemical and structural corroboration of the functional phenotype.

**Conclusion:**

We describe the first primary isolated heart model reproducing the core functional phenotype of TTS entirely within an isolated heart preparation. Thus, intrinsic myocardial responses to acute catecholaminergic stress are sufficient to generate regional dysfunction of TTS in the absence of systemic neurohumoral input.

## Introduction

Takotsubo syndrome (TTS) is an acute cardiac condition characterized by transient regional left ventricular dysfunction, typically manifesting as extensive apical akinesia, that mimics acute myocardial infarction in the absence of obstructive coronary artery disease.^1–6^ Although frequently triggered by emotional or physical stress and associated with catecholamine excess, the underlying mechanisms remain incompletely understood.^7–12^ Progress in elucidating TTS pathophysiology has been hindered by limitations of current experimental models, which often lack reproducibility and fail to isolate primary myocardial responses from systemic neurohormonal and haemodynamic influences.^13–22^ These constraints limit our ability to interpret intrinsic myocardial responses and hinder the identification of appropriate therapeutic targets. Consequently, there is a pressing need for reductionist models that enable precise mechanistic dissection at the organ level and support preclinical therapy development.^23–26^

To address this gap, we developed a primary isolated heart TTS model, defined as a preparation in which the TTS-like phenotype is induced entirely within the *ex vivo* heart, without prior *in vivo* catecholamine exposure. Using a Langendorff-perfused rat heart preparation with controlled intra-aortic isoprenaline administration, we replicated hallmark features of TTS, including systolic dysfunction, diastolic impairment, and regional wall-motion abnormalities, in the complete absence of systemic neurohormonal and other extrinsic inputs.^1,20,22,27^ This platform affords precise experimental control, enabling real-time monitoring of myocardial function and high-resolution characterization of disease evolution. It thus establishes a standardized, reproducible framework for investigating TTS pathophysiology and for testing candidate therapies under defined conditions.^10,28^

## Methods

### Study design and experimental groups

Twenty male Sprague–Dawley rats (4–6 weeks of age, ∼250 g) were randomly allocated to one of two experimental groups: control (*n* = 10), perfused with Krebs–Henseleit buffer alone, and **primary isolated heart TTS** (*n* = 10), receiving intra-aortic isoprenaline infusion to induce catecholamine-mediated myocardial dysfunction. An additional **secondary isolated heart TTS** group (*n* = 6), consisting of hearts isolated from rats with previously established *in vivo* Takotsubo syndrome,^22^ was included exclusively for echocardiographic comparison and was not incorporated into haemodynamic, statistical, or mechanistic analyses. Animals were randomly assigned to experimental groups using blinded allocation, and all outcome assessments were performed by investigators blinded to group allocation.

### Langendorff perfusion

Following anaesthesia (ketamine 75 mg/kg and midazolam 5 mg/kg, intraperitoneally) and systemic heparinization (500–1000 IU/kg), hearts were rapidly excised and mounted on a Langendorff perfusion apparatus (LSC070 MDE, Hungary) operating in constant-pressure mode. Total warm ischaemic time was maintained below 75 s. Hearts were perfused with oxygenated Krebs–Henseleit buffer (mmol/L: NaCl 118, KCl 4.7, MgSO₄ 1.2, KH₂PO₄ 1.2, NaHCO₃ 25, CaCl₂ 1.8, glucose 11) equilibrated with 95% O₂/5% CO₂, maintained at pH 7.40 and 37.5°C. Baseline coronary flow was maintained between 12 and 15 mL/min.

### Isoprenaline administration

To induce the primary isolated heart TTS phenotype, isoprenaline hydrochloride (Sigma–Aldrich) was infused directly into the aortic cannula through a dedicated side port over 15 min. The total administered dose corresponded to 1 mg/kg body weight, matching the dose used in our previously validated *in vivo* model.^22^ Because the isolated-heart preparation lacks systemic distribution and metabolism, the infusion rate was adjusted to achieve a final coronary perfusate concentration of ∼5 μM (range 4.5–5.6 μM), calculated from the measured coronary flow (12–15 mL/min). This concentration was selected because it consistently produced the characteristic isolated-heart TTS phenotype under the experimental conditions used in the present study. Control hearts received vehicle infusion only. Haemodynamic variables were recorded continuously throughout the subsequent 6-h perfusion period. Hearts comprising the secondary isolated heart TTS group underwent *in vivo* induction using our previously established protocol,^22^ consisting of intravenous tail-vein infusion of isoprenaline (1 mg/kg over 15 min), followed by confirmation of the TTS phenotype using high-resolution transthoracic echocardiography prior to heart isolation.

### Haemodynamic measurements

Left ventricular pressure was continuously recorded using a saline-filled intraventricular latex balloon positioned within the left ventricular cavity. Balloon volume was adjusted at baseline to achieve a stable left ventricular end-diastolic pressure (LVEDP) of 6–10 mmHg and was maintained unchanged throughout the experiment to ensure comparable preload conditions across all preparations. Directly measured haemodynamic variables included heart rate (HR), left ventricular systolic pressure (LVSP), LVEDP, left ventricular developed pressure (LVDP), maximum and minimum rates of left ventricular pressure development (dP/dt_max and dP/dt_min), and coronary flow (CF), which was measured continuously using an inline flow probe integrated within the perfusion circuit.

Derived haemodynamic variables were calculated to characterize myocardial workload, coronary vascular function, and contractile-relaxation dynamics. Rate-pressure product (RPP) was calculated as HR × LVDP and used as an index of myocardial mechanical workload. Relative RPP (rRPP) was expressed as the percentage of each heart's baseline RPP. Coronary vascular resistance (CVR) was calculated as perfusion pressure divided by coronary flow (mmHg·min/mL). The isovolumic relaxation constant (TAU) was not obtained by direct exponential fitting of ventricular pressure decay. Instead, a predefined surrogate estimate was applied uniformly to all recordings using the empirically derived relationship between the magnitude of dP/dt_min and ventricular relaxation: **τ (ms)**  **=**  **−3.50**  **×**  **ln(|dP/dt_min|)**  **+**  **35.00**. This standardized analytical approach was applied identically to every heart and every time point, providing a consistent relative index of diastolic relaxation across the dataset. Accordingly, all reported TAU values represent this predefined surrogate estimate rather than directly fitted relaxation constants. Estimated developed tension (eDT) was calculated from LVDP using a simplified form of the Law of Laplace under the predefined assumptions of constant ventricular radius and wall thickness. Because these assumptions may not remain strictly valid during development of regional wall-motion abnormalities and apical ballooning, eDT was not interpreted as a direct measure of myocardial wall tension but rather as an exploratory comparative index of contractile behaviour. All derived variables were calculated using predefined equations and independently verified for mathematical consistency before statistical analysis.

### Echocardiography

Cardiac imaging was performed using a Vevo 3100 high-resolution ultrasound system equipped with a 21-MHz transducer. Hearts were imaged in the parasternal long-axis view through a temperature-controlled water bath. Regional wall-motion abnormalities, apical ballooning, and mechanical dyssynchrony were assessed qualitatively, while speckle-tracking strain analysis was used to quantify global longitudinal strain (GLS), left ventricular akinesia index (LVAI), and segmental time-to-peak strain dispersion. Strain values are reported using the conventional negative scale, with more negative GLS indicating better systolic function.

Because echocardiography was performed in isolated Langendorff-perfused hearts under unloaded isovolumic conditions, conventional indices of systolic performance, including ejection fraction (EF), were interpreted as relative functional indices rather than direct measures of physiological ventricular pumping capacity. EF was therefore measured both immediately before isoprenaline administration and after completion of the 6-h perfusion protocol to quantify relative changes in ventricular function within an identical experimental preparation. All echocardiographic measurements were performed independently by two investigators blinded to experimental group, and any discrepancies were resolved by consensus.

### Phenotype definition

The primary isolated heart TTS phenotype was defined by the simultaneous presence of^1^ regional apical or mid-ventricular akinesia involving >20% of the left ventricular endocardial length with relative preservation of basal contraction;^2^ characteristic apical ballooning on parasternal long-axis echocardiography;^3^ reduced ejection fraction relative to control hearts; and^4^ mechanical dyssynchrony demonstrated by increased segmental time-to-peak strain dispersion. These criteria were adapted from our previously validated *in vivo* model^22^ and are consistent with the characteristic imaging features described in contemporary clinical diagnostic frameworks for Takotsubo syndrome.^2,14^

### Biochemical corroboration

To provide biochemical corroboration of myocardial injury, coronary effluent samples were collected from a subset of control and primary isolated TTS hearts at predefined time points during the perfusion protocol (0, 5, 10, 15, 30, 60, and 90 min after the start of perfusion). Cardiac troponin I (cTnI) and cardiac troponin T (cTnT) concentrations were measured using clinically validated immunoassays according to the manufacturers’ instructions. cTnT concentrations were measured using the Roche Cobas assay, with a limit of blank (LoB) of 3 ng/L, a limit of quantification (LoQ) of 5 ng/L, an analytical coefficient of variation (CV) of 5%, and a 99th percentile reference value of 14 ng/L. cTnI levels were determined using the Abbott Alinity assay, with an LoB of 1 ng/L, an LoQ of 5.1 ng/L, an analytical CV of 5%, and a 99th percentile reference value of 26 ng/L.

### Histological corroboration

To provide structural corroboration of the functional phenotype, representative control and primary isolated TTS hearts were collected at the completion of the perfusion protocol for routine histological evaluation. Hearts were fixed in neutral-buffered formalin, paraffin embedded, sectioned, and stained with haematoxylin and eosin (H&E) and Masson's trichrome using standard laboratory protocols. Histological assessment was performed descriptively to evaluate overall myocardial architecture and identify representative structural alterations associated with the isolated-heart TTS phenotype.

### Statistical analysis

The primary endpoint was the group-by-time interaction for LVDP over the 360-min perfusion period, analysed using generalized additive mixed models (GAMMs) with experimental group as a fixed effect, group-specific smooth functions for time, and random intercepts for individual rats. Key secondary endpoints (rate-pressure product, coronary flow, and TAU) were analysed using the same modelling strategy. False discovery rate correction (Benjamini–Hochberg) was applied across these four confirmatory endpoints (*q* < 0.05). Prespecified exploratory analyses were performed to characterize temporal evolution of the phenotype and multivariate relationships within the dataset. These included principal component analysis (PCA) with PERMANOVA (999 permutations; rat-stratified), Gaussian mixture modelling to identify latent physiological states, hierarchical clustering, XGBoost classification with SHAP (SHapley Additive exPlanations)-based feature interpretation, and exploratory directional time-series analyses.^29^ For integrated haemodynamic–echocardiographic PCA, highly collinear variables were reduced before modelling to improve component interpretability. Exploratory directional analyses were performed after resampling group-mean trajectories to regular 15-min intervals, followed by standardization and first differencing. Analyses were restricted to directly measured haemodynamic variables to avoid mathematical coupling introduced by derived indices. Pairwise tests were corrected using the Benjamini–Hochberg false discovery rate procedure. No directional relationship remained significant after multiple-testing correction; accordingly, these analyses were interpreted as exploratory and were not used to support mechanistic or causal inference. All machine-learning and multivariate analyses were considered exploratory and hypothesis-generating. Detailed descriptions of exploratory analyses, cross-validation procedures, sensitivity analyses, and implementation are provided in the Supplementary Methods. Sample size (*n* = 10 per group) was determined *a priori* from pilot data showing a between-group difference in LVDP at 360 min (partial η^2^ = 0.19; α = 0.05; power = 0.80). Statistical analyses were performed using Python (v3.11) and R (v4.3.1). Source code is provided in the Supplementary material.

## Results

### Haemodynamic characterization of primary isolated heart TTS

Twelve physiological parameters were continuously monitored over 360 min (37 time points) in the control (*n* = 10) and primary isolated heart TTS (*n* = 10) groups (*Figure 1*). Control hearts maintained stable function throughout the 6-h perfusion period. In contrast, primary isolated TTS hearts exhibited progressive deterioration of LVDP, RPP, and contractile indices (dP/dt_max, dP/dt_min), accompanied by prolongation of the isovolumic relaxation constant (TAU, where TAU values were calculated using the predefined surrogate method described in the Methods and are interpreted as relative indices of ventricular relaxation that was applied uniformly across all animals and time points) and a progressive reduction in coronary flow, reflecting coordinated deterioration of contractile, relaxation, and perfusion-related indices (*Figure 2*, *Table 1*). The primary endpoint, LVDP group-by-time interaction, was highly significant (GAMM: *P* < 0.001; *q* = 0.002). Key secondary endpoints similarly demonstrated significant group-by-time effects after FDR correction: RPP (*q* = 0.006), TAU (*q* = 0.001), and CF (*q* = 0.005). Effect sizes were substantial (LVDP: partial η^2^ = 0.47; RPP: partial η^2^ = 0.42) (see Supplementary material online, *Figure S1*).

**Figure 1:**
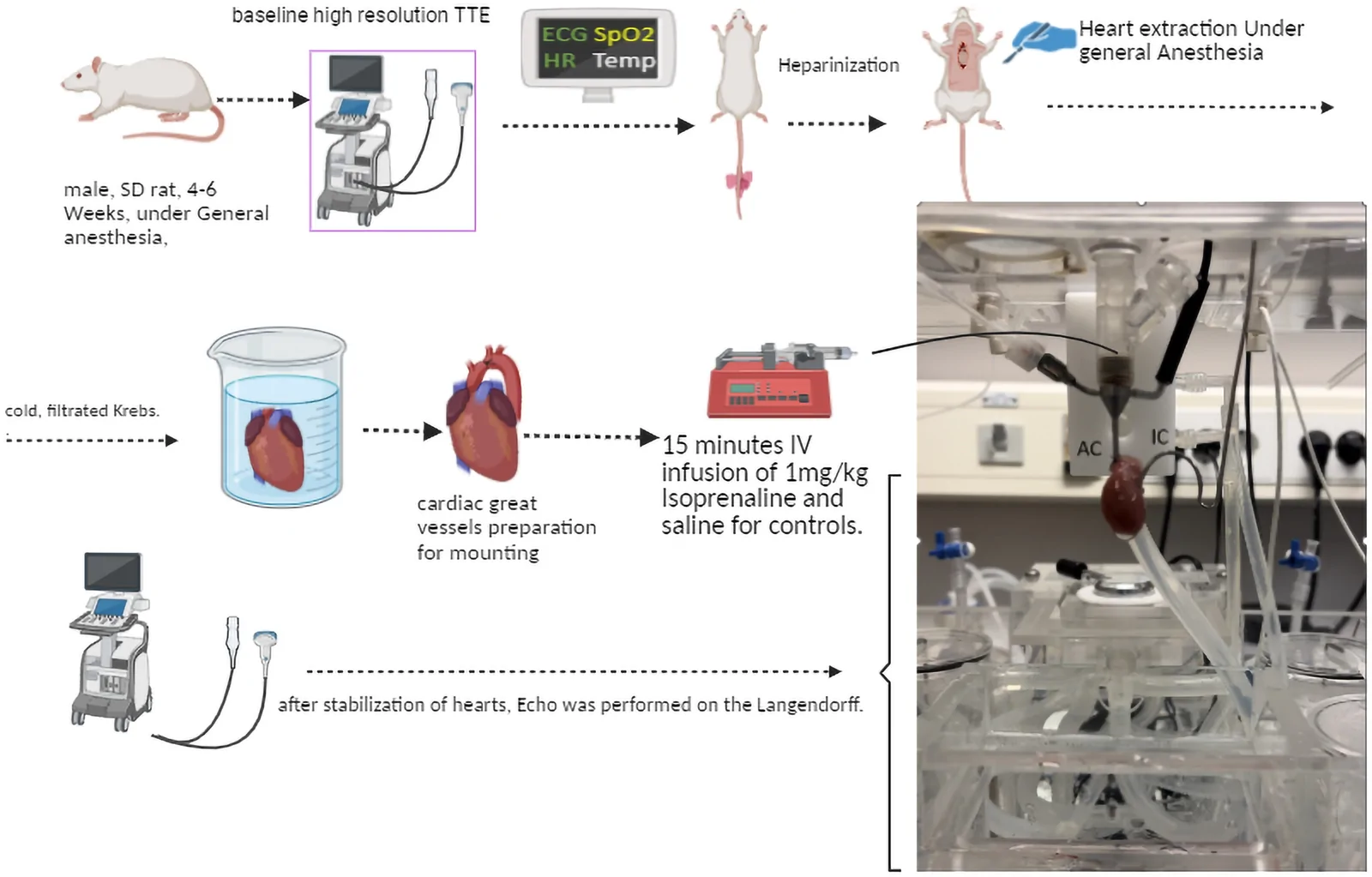
Schematic of the primary isolated heart TTS model. Langendorff perfusion system with constant-pressure perfusion, intra-aortic isoprenaline infusion port, and high-resolution ultrasound imaging for real-time functional assessment. Hearts were monitored continuously for 6 h following catecholamine exposure.

**Figure 2:**
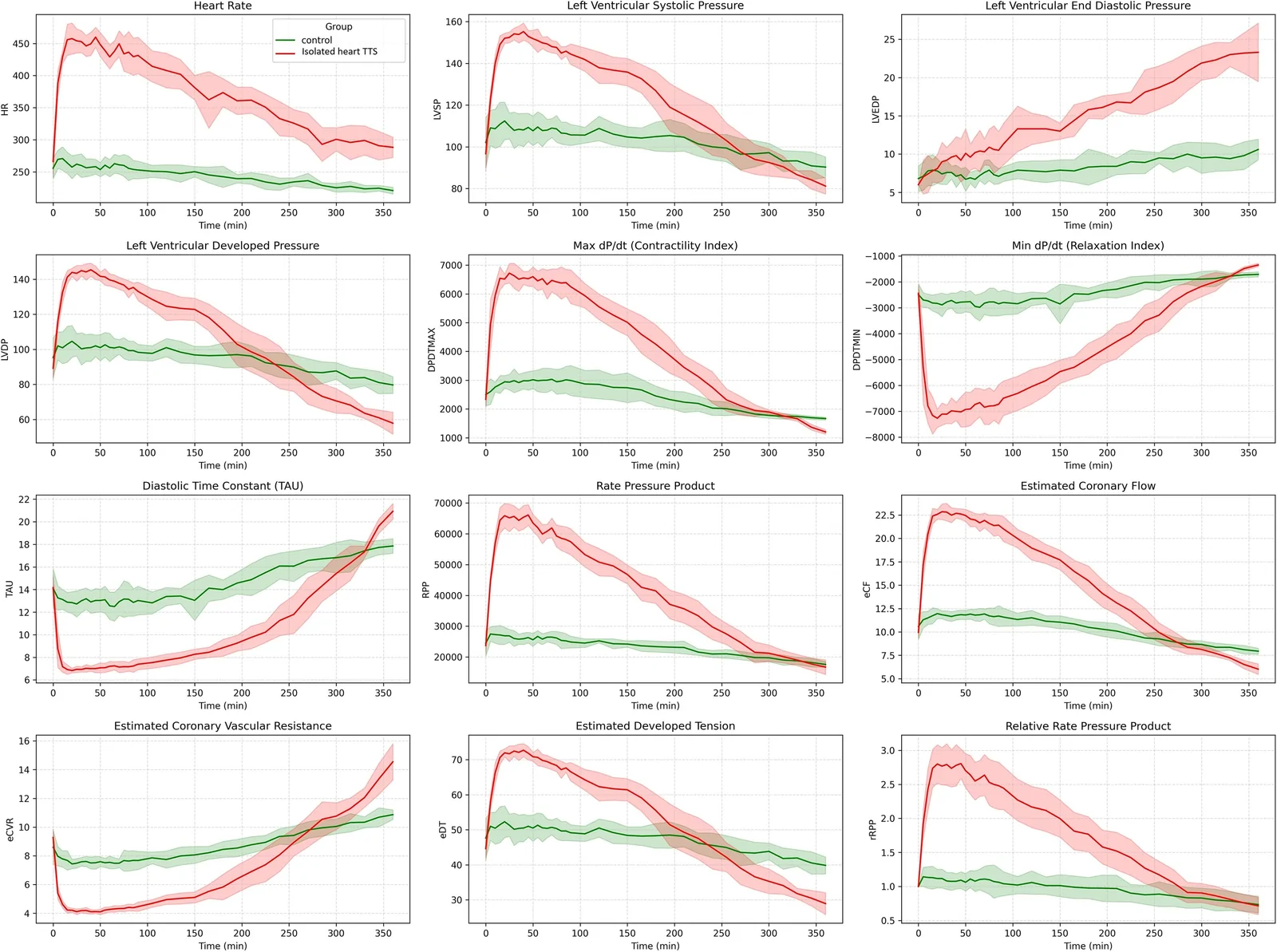
Comprehensive haemodynamic characterization of the primary isolated heart TTS phenotype. Time-series trajectories (mean ± 95% confidence intervals) of 12 continuously recorded haemodynamic parameters over 360 min in control (*n* = 10) and primary isolated heart TTS (*n* = 10) hearts. Following intra-aortic isoprenaline administration, primary isolated heart TTS hearts exhibited an initial hyperdynamic response followed by progressive deterioration of systolic function, diastolic relaxation, myocardial work, and coronary perfusion compared with controls, illustrating coordinated evolution of the TTS-like phenotype. Abbreviations: HR, heart rate; LVSP, left ventricular systolic pressure; LVEDP, left ventricular end-diastolic pressure; LVDP, left ventricular developed pressure; dP/dtmax, maximum rate of pressure development; dP/dtmin, minimum rate of pressure decline; TAU, isovolumic relaxation constant (calculated using the predefined surrogate described in Methods); RPP, rate-pressure product; CF, coronary flow; CVR, coronary vascular resistance; eDT, estimated developed tension; rRPP, relative rate-pressure product.

**Table 1:** Summary of key haemodynamic and echocardiographic parameters at baseline and 360 min in control and primary isolated heart Takotsubo syndrome hearts

Parameter	Control (baseline)	Control (360 min)	TTS (baseline)	TTS (360 min)	*P*-value
LVDP (mmHg)	110 ± 5	108 ± 4	112 ± 6	68 ± 8	<0.001
RPP (mmHg·bpm)	28 000 ± 1200	27 500 ± 1100	28 500 ± 1300	18 000 ± 1500	<0.001
TAU (ms)	20 ± 2	21 ± 2	20 ± 2	35 ± 4	0.001
CF (mL/min)	14 ± 1	13 ± 1	14 ± 1	10 ± 1	0.005
EF (%)	65 ± 3	64 ± 3	66 ± 3	45 ± 5	<0.01
GLS (%)	−20 ± 1	−19 ± 1	−21 ± 1	−14 ± 2	<0.01

Summary of key haemodynamic and echocardiographic findings at baseline and 360 min in six control and primary isolated heart TTS groups (*n* = 10/group). Parameters include left ventricular developed pressure (LVDP), rate-pressure product (RPP), isovolumic relaxation constant (TAU), coronary flow (CF, measured), ejection fraction (EF), and global longitudinal strain (GLS), with *P*-values indicating significant differences in TTS hearts at 360 min.

### Triphasic trajectory of functional decline

GAMM analysis revealed a consistent triphasic trajectory in primary isolated heart TTS (*Figure 3*). The hyperactivation phase (0–60 min) was characterized by elevated heart rate, LVDP, and RPP, reflecting the acute catecholamine response. The transition phase (60–240 min) showed fluctuating contractile performance with progressive diastolic impairment, evidenced by rising TAU. The functional nadir phase (240–360 min) was marked by diminished contractile performance, reduced coronary perfusion, and maximal haemodynamic impairment, with partial recovery observed towards the end of the monitoring period. First-derivative analysis of GAMM smooths identified inflection points at ∼60 and 240 min, corresponding to the boundaries between the three haemodynamic phases (*Figure 4*, Supplementary material online, *F*igure  *S2*).

**Figure 3:**
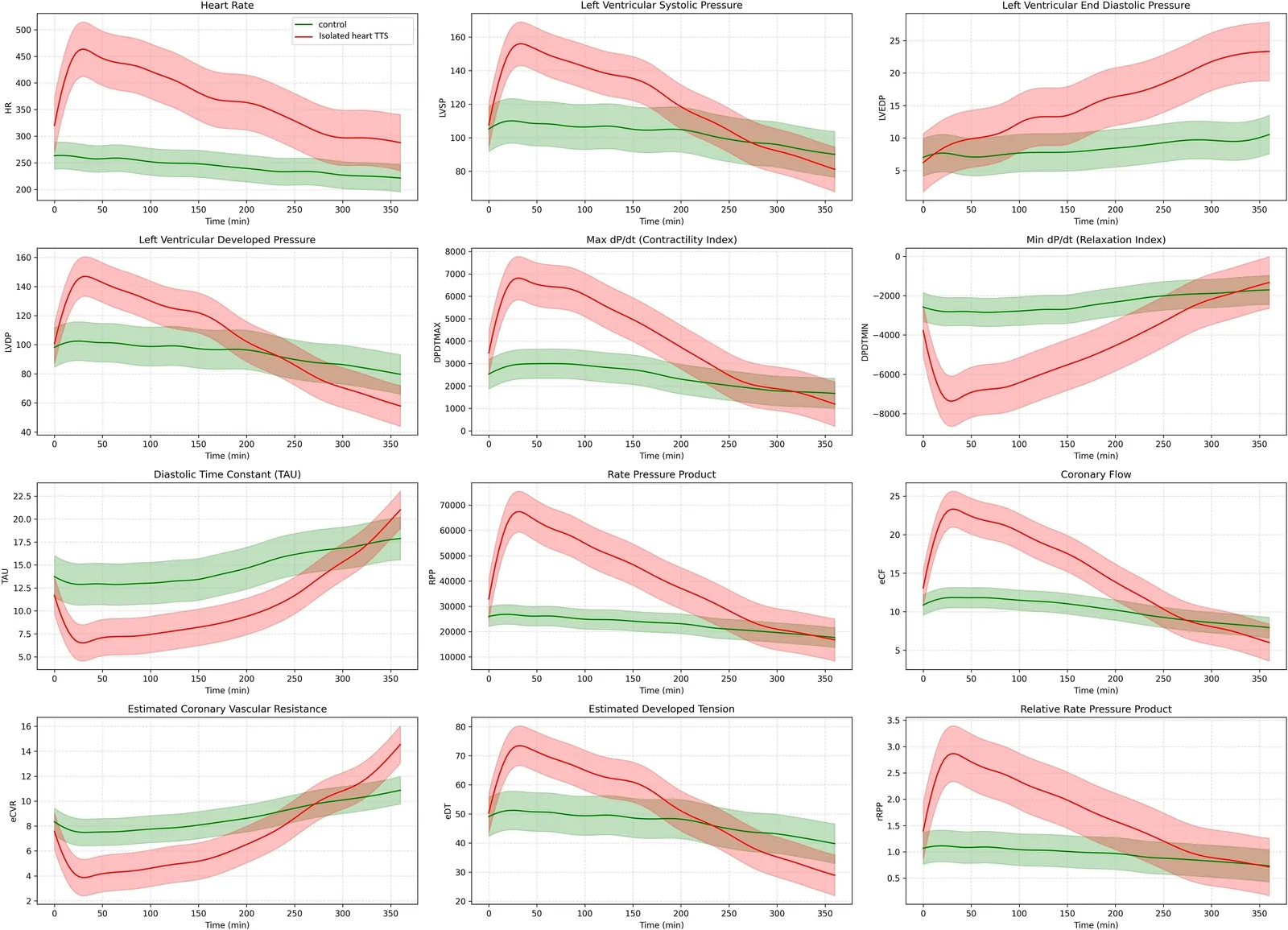
Generalized additive mixed model (GAMM) analysis demonstrates the triphasic evolution of the primary isolated heart TTS phenotype. GAMM-fitted trajectories (mean ± 95% confidence intervals) for heart rate (HR), left ventricular developed pressure (LVDP), isovolumic relaxation constant (TAU), and coronary flow (CF) in control (green, *n* = 10) and primary isolated heart TTS (red, *n* = 10). The fitted trajectories identify three reproducible physiological phases following catecholamine exposure: an initial hyperactivation phase (0–60 min), a transition phase (60–240 min), and a functional nadir phase (240–360 min), characterized by progressive impairment of contractility, relaxation, and coronary perfusion. The group-by-time interaction was significant for all parameters shown.

**Figure 4:**
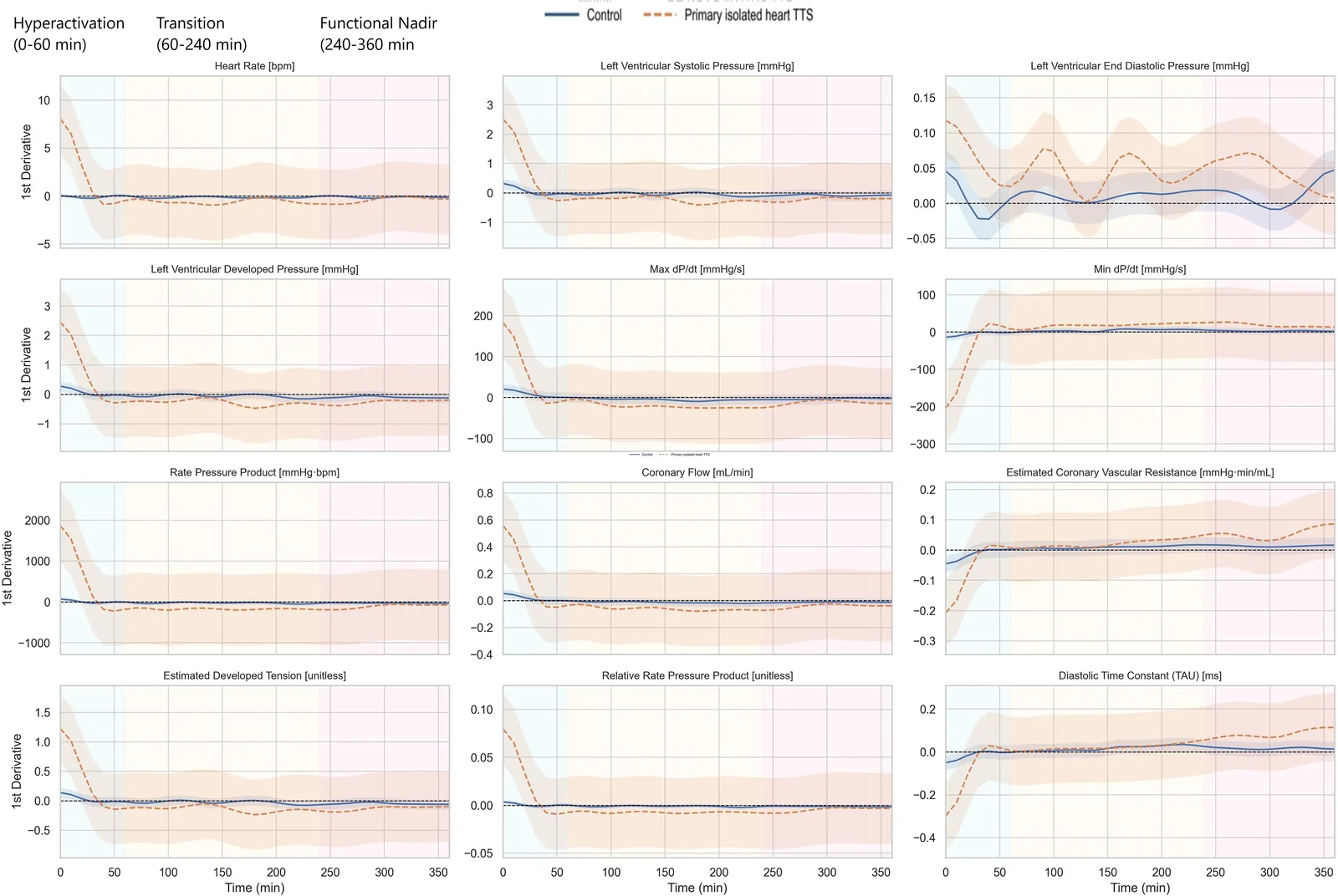
First-derivative analysis of GAMM-fitted trajectories identifies temporal changes in the primary isolated heart TTS phenotype. First derivatives of the fitted GAMM smooths for heart rate, left ventricular developed pressure, isovolumic relaxation constant (TAU), and coronary flow in control and primary isolated TTS hearts. Solid blue lines represent control hearts, and dashed orange lines represent primary isolated TTS hearts; shaded regions indicate 95% confidence intervals. Positive derivative values indicate an increasing fitted trajectory, negative values indicate a decreasing trajectory, and crossings of zero indicate changes in trajectory direction. Background regions denote the predefined hyperactivation (0–60 min), transition (60–240 min), and functional nadir (240–360 min) phases. Coordinated changes across the four physiological domains supported approximate transition points at 60 and 240 min. The derivative analyses were exploratory and were used to characterize temporal patterning rather than establish biological mechanisms.

### Multivariate group separation

Principal component analysis demonstrated clear separation between control and primary isolated heart TTS trajectories in low-dimensional space (see Supplementary material online, *Figure S2*). PC1 captured metabolic and contractile parameters, while PC2 reflected diastolic function and vascular resistance. Exploratory PERMANOVA showed significant group separation (*P* < 0.001, η^2^ = 0.36; rat-stratified permutations). An XGBoost classifier distinguished groups with an ROC AUC of 0.92 (95% CI: 0.88–0.96) using leave-one-rat-out cross-validation. SHAP analysis identified heart rate, coronary vascular resistance, and LVEDP as the most discriminative features, with temporal SHAP trajectories showing that early discrimination relied on heart rate, then shifted to TAU and CF in later phases (see Supplementary material online, Figures  *S3* and *S4*).

### Physiological state clustering

Gaussian mixture modelling identified three latent physiological states corresponding to the hyperactivation, transition, and functional nadir phases (see Supplementary material online, *Figures S5* and *S6*). Model selection favoured *k* = 3 clusters (ΔBIC ≥6 vs. *k* = 2 or *k* = 4; bootstrap stability with mean Jaccard index ≥0.75). Cluster transitions aligned temporally with GAMM-derived inflection points and shifts in SHAP feature importance, detecting the robustness of the triphasic framework. Overlay of cluster assignments onto individual parameter trajectories confirmed consistent phase alignment across all 12 monitored variables (see Supplementary material online, *Figures S5* and *S6*).

### Cross-correlation and predictive relationships

Cross-correlation analysis revealed a strong positive association between CF and LVDP (*r* = 0.83, *P* < 0.001) and an inverse association between TAU and dP/dt_max (*r* = −0.74, *P* < 0.001), indicating close temporal coupling between perfusion, contractile performance, and relaxation dynamics (*Table 2*). Exploratory directional analyses restricted to directly measured variables did not identify relationships that remained significant after correction for multiple comparisons. Accordingly, the observed temporal patterns are interpreted as coordinated associations accompanying phenotype progression rather than evidence that one haemodynamic variable mechanistically or predictively drives another. These findings should be interpreted as predictive temporal associations only and do not establish whether, for example, reduced CF contributed directly to functional deterioration, reflected secondary myocardial dysfunction, or evolved bidirectionally with the haemodynamic phenotype. Exploratory hierarchical clustering separated control and primary isolated TTS hearts according to their integrated haemodynamic profiles and identified correlation-based groupings of contractile/workload and diastolic/vascular variables (see Supplementary material online, *Figure S7*).

**Table 2:** Cross-correlation analysis of haemodynamic variables demonstrating temporal associations among contractile, relaxation, and perfusion-related parameters during progression of the primary isolated heart Takotsubo syndrome phenotype

	HR	LVSP	LVEDP	LVDP	DPDTMAX	DPDTMIN	RPP	TAU	eCF	eCVR	eDT	rRPP
**HR**	1	0.93	−0.25	0.93	0.94	−0.49	0.97	−0.35	0.95	−0.39	0.93	0.97
**LVSP**	0.93	1	−0.25	0.99	0.97	−0.54	0.98	**−**0.43	0.98	**−**0.48	0.99	0.98
**LVEDP**	−0.25	**−**0.25	1	**−**0.28	**−**0.3	0.91	**−**0.29	0.88	**−**0.3	0.89	**−**0.28	**−**0.29
**LVDP**	0.93	0.99	−0.28	1	0.97	**−**0.55	0.98	**−**0.39	0.99	**−**0.46	1	0.98
**DPDTMAX**	0.94	0.97	**−**0.3	0.97	1	**−**0.56	0.97	**−**0.37	1	**−**0.44	0.97	0.97
**DPDTMIN**	**−**0.49	**−**0.54	0.91	**−**0.55	**−**0.56	1	**−**0.54	0.95	**−**0.56	0.95	**−**0.55	**−**0.54
**RPP**	0.97	0.98	**−**0.29	0.98	0.97	**−**0.54	1	**−**0.36	0.99	**−**0.43	0.98	1
**TAU**	**−**0.35	**−**0.43	0.88	**−**0.39	**−**0.37	0.95	**−**0.36	1	**−**0.38	0.98	**−**0.39	**−**0.36
**eCF**	0.95	0.98	**−**0.3	0.99	1	**−**0.56	0.99	**−**0.38	1	**−**0.44	0.99	0.99
**eCVR**	**−**0.39	**−**0.48	0.89	**−**0.46	**−**0.44	0.95	**−**0.43	0.98	**−**0.44	1	**−**0.46	**−**0.43
**eDT**	0.93	0.99	**−**0.28	1	0.97	**−**0.55	0.98	**−**0.39	0.99	**−**0.46	1	0.98
**rRPP**	0.97	0.98	**−**0.29	0.98	0.97	**−**0.54	1	**−**0.36	0.99	**−**0.43	0.98	1

Cross-correlation analysis of physiological parameters in primary isolated heart TTS (*n* = 10/group), showing correlation coefficients (*r*) for key parameter pairs, including coronary flow (CF, measured) with left ventricular developed pressure (LVDP) and isovolumic relaxation constant (TAU) with dP/dt_max.

### Echocardiographic validation

Echocardiographic assessment performed following completion of the 6-h perfusion protocol confirmed the development of a TTS-like phenotype in primary isolated TTS hearts (*Figure 5*). Representative echocardiographic images obtained during diastole and systole illustrate preservation of normal chamber geometry and systolic contraction in control hearts, whereas primary and secondary isolated TTS hearts expressed characteristic apical ballooning, defined here as akinesia of the apical segments with relative preservation of basal contraction, and referred to as apical ballooning hereafter (*Figure 5*).

**Figure 5:**
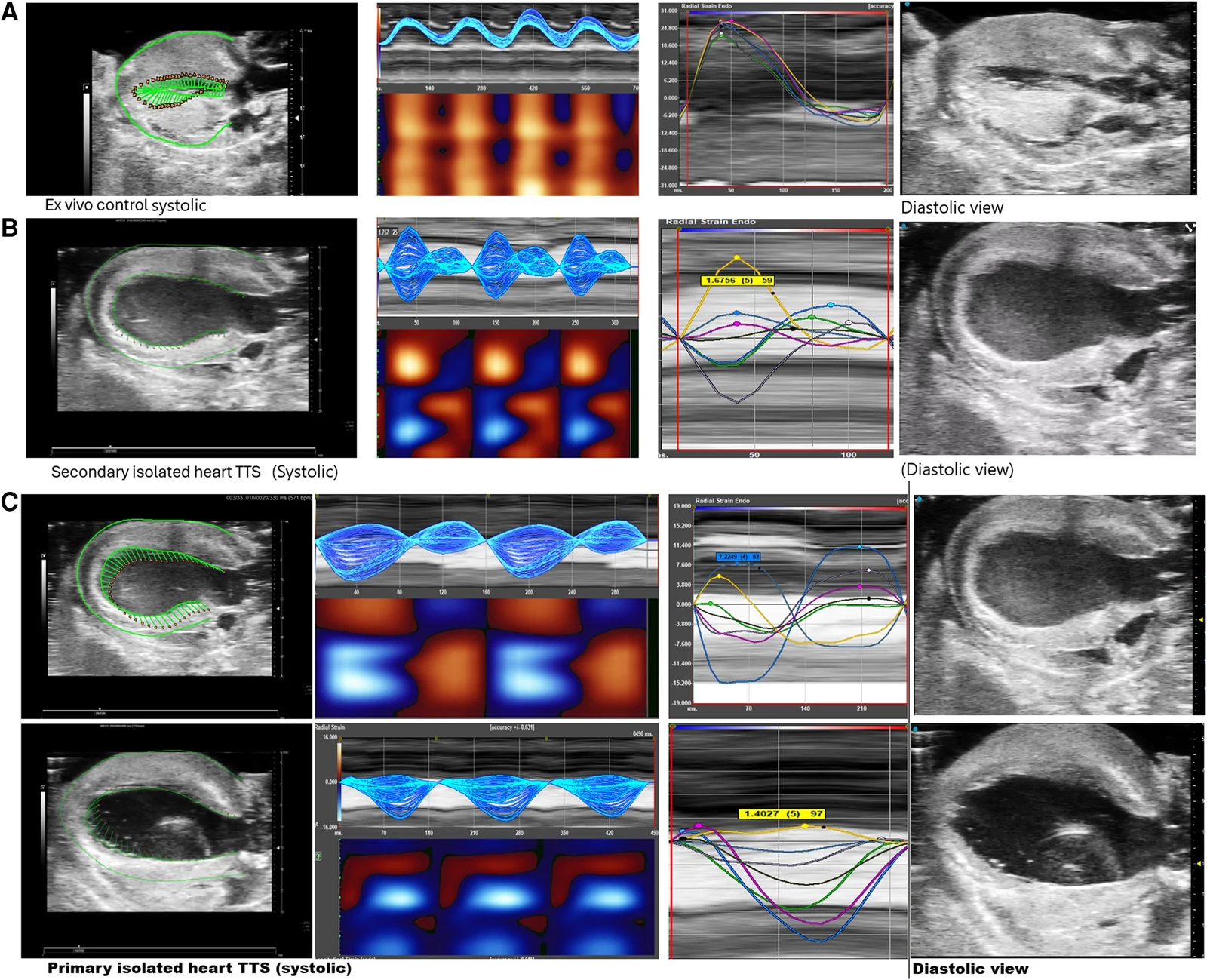
Representative echocardiographic cine loops demonstrating apical ballooning. High-resolution echocardiography with speckle-tracking strain analysis in parasternal long-axis view. (*A*) Control heart showing synchronous contraction with uniform strain distribution. (*B*) Secondary isolated heart TTS (*in vivo* induction, *ex vivo* perfusion) demonstrating mechanical dyssynchrony and regional wall-motion abnormality. (*C*) Primary isolated heart TTS showing characteristic apical akinesia, mechanical dyssynchrony, and regional wall-motion abnormality, the hallmark TTS phenotype induced entirely within the isolated preparation.

Baseline echocardiographic assessment exhibited comparable EF values across all experimental groups before isoprenaline administration, indicating similar initial functional status of the isolated heart preparations (*Figure 6A–C*). Following completion of the 6-h perfusion protocol, control hearts exhibited only a modest reduction in EF, whereas both primary and secondary isolated TTS hearts demonstrated a substantially greater decline in EF. Because all hearts were examined under identical unloaded Langendorff conditions, these changes are interpreted as relative differences in functional performance within the experimental preparation rather than direct measures of physiological ventricular pumping capacity.

**Figure 6:**
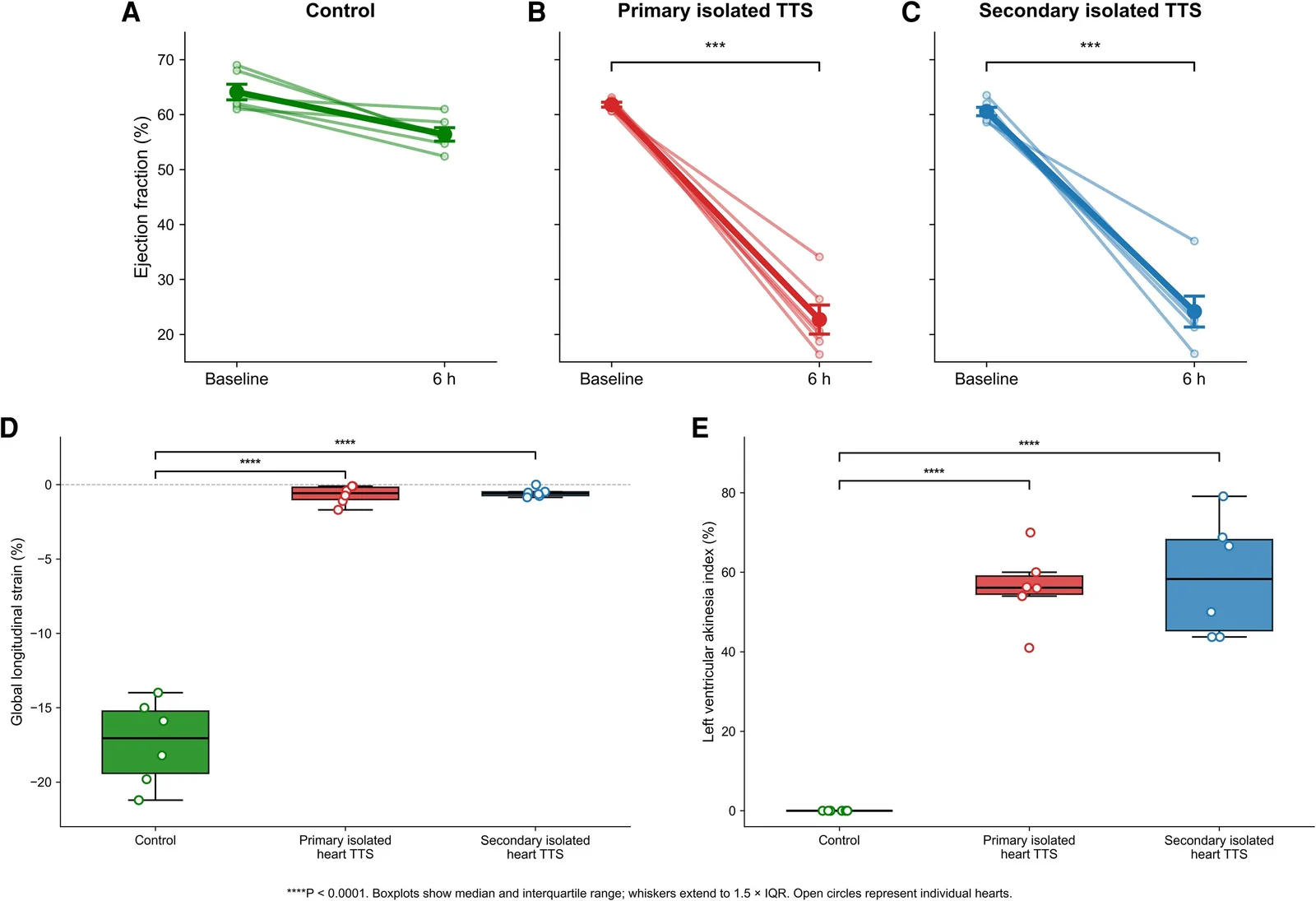
Quantitative echocardiographic characterization of the catecholamine-induced TTS-like phenotype. (*A–C*) Individual changes in ejection fraction (EF) from baseline to 6 h in control, primary isolated heart TTS, and secondary isolated heart TTS preparations, respectively. Thin lines represent individual hearts, and thick lines with error bars represent group mean ± SEM (*n* = 6 per group). Baseline EF was comparable across groups; control hearts showed a modest decline over 6 h, whereas both TTS groups demonstrated marked reductions in EF. ***P < 0.001 vs. baseline within the same group using (Annova). (*D*) Global longitudinal strain (GLS) and (*E*) left ventricular akinesia index (LVAI) at 6 h in control, primary isolated heart TTS, and secondary isolated heart TTS hearts. Boxplots show median and interquartile range, and superimposed points represent individual hearts. Less negative GLS values indicate impaired systolic deformation. Both TTS groups exhibited less negative GLS and increased LVAI compared with control hearts, demonstrating impaired global systolic deformation together with regional apical dysfunction. Statistical comparisons were performed using Annova and Tukey post hoc procedure.

Consistent with these findings, primary isolated TTS hearts exhibited significantly less negative GLS, indicating impaired systolic deformation, together with a marked increase in LVAI, reflecting characteristic apical hypokinesis despite preserved basal contraction (all *P* < 0.0001; *Figure 6D* and *E*, *Table 1*, Supplementary material online, *Table S1*).

To further contextualize these observations, echocardiographic findings were compared with those from a secondary group of isolated hearts from rats with prior *in vivo* TTS induction, subsequently studied *ex vivo* (*n* = 6). Both TTS groups compared exhibited closely comparable echocardiographic abnormalities relative to controls, including reduced EF, impaired GLS, elevated LVAI, and increased segmental strain heterogeneity, particularly in the longitudinal plane (*Figure 6*, Supplementary material online, *Figure S8*). This concordance supports the reproducibility of the TTS-like echocardiographic phenotype observed in the primary isolated heart model. Correlation analysis demonstrated consistent relationships between invasive haemodynamic deterioration and echocardiographic indices of systolic dysfunction (see Supplementary material online, *Figure S9*), identifying concordance between the two complementary assessment modalities.

Combined principal component analysis integrating haemodynamic and echocardiographic variables explained 89.2% of the total variance, with clear separation between control and primary isolated heart TTS groups. PC1 accounted for 77.4% of the variance and was primarily driven by contractile and perfusion-related variables, including RPP, LVSP, LVDP, and coronary flow, whereas PC2 accounted for 11.8% of the variance and was predominantly influenced by diastolic and strain-derived variables, particularly LVAI and GLS (see Supplementary material online, Figure 10, Supplementary material online, *Table S2*). Moreover, *Table 3* provides an integrated overview of the principal haemodynamic, echocardiographic, clustering, and statistical findings, summarizing the major physiological determinants of the primary isolated heart TTS phenotype.

**Table 3:** Integrated summary of the principal haemodynamic, echocardiographic, clustering, and statistical findings characterizing the primary isolated heart Takotsubo syndrome phenotype

Parameter	*P*-value (FDR)	*q*-value (FDR)	Time of change (min)	SHAP Rank	GMM cluster	Echo correlation
LVDP	<0.001	0.002	240	1	Functional nadir	LVAI% (*r* = 0.64)
RPP	<0.001	0.006	60	2	Hyperactivation	Radial strain (*r* = −0.50)
TAU	<0.001	0.001	100	3	Transition	GLS% (*r* = −0.72)
CF	<0.005	0.005	60	4	Functional nadir	None
DPDTMAX	<0.01	0.010	120	5	Transition	GLS% (*r* = −0.60)
HR	<0.05	0.045	60	6	Hyperactivation	EF% (*r* = 0.68)
LVEDP	<0.01	0.010	180	7	Functional nadir	LVAI% (*r* = 0.64)
eCVR	<0.01	0.010	120	8	Transition	None
eDT	<0.05	0.045	240	9	Functional nadir	GLS% (*r* = −0.55)
DPDTMIN	<0.05	0.045	120	10	Transition	None
rRPP	<0.01	0.010	180	11	Functional nadir	LVAI% (*r* = 0.64)
LVSP	<0.05	0.045	60	12	Hyperactivation	EF% (r = 0.68)

Integrative matrix linking statistical significance, SHAP (Shapley Additive Explanations) feature importance, phase-specific activation, Gaussian Mixture Model (GMM) clustering, and echocardiographic correlations across 12 key physiological parameters (e.g. LVDP, RPP, TAU, CF, HR, LVEDP) in primary isolated heart TTS (*n* = 10/group). The matrix provides a comprehensive overview of phenotypic dynamics and mechanistic drivers. *P*-values are corrected for multiple testing using false discovery rate (FDR); *q*-values represent adjusted *P*-values under FDR correction (*q* < 0.05 indicates significance). The echo correlation column lists significant echocardiographic parameters with Pearson correlation coefficients (*r*) where applicable (*P* < 0.05); ‘None’ indicates no significant correlation (*P* ≥ 0.05). Gaussian mixture models (*k* = 2–6) were compared by BIC; *k* = 3 minimized BIC (ΔBIC ≥ 6 vs. *k* = 2.4) with bootstrap stability (mean Jaccard ≥ 0.75). The three states aligned with hyperactivation, transition/instability, and functional nadir.

### Biochemical and structural corroboration of the functional phenotype

To provide supportive biochemical corroboration of the observed functional phenotype, cTnI and cTnT concentrations were measured serially in coronary effluent collected from a subset of control and primary isolated TTS hearts during the early phase of the perfusion protocol (see Supplementary material online, Figure 11). Both biomarkers remained comparatively stable throughout the sampling period in control hearts, whereas primary isolated TTS hearts demonstrated a progressive increase in both cTnI and cTnT concentrations following isoprenaline administration. Although only modest differences were observed immediately following the infusion period, biomarker concentrations diverged progressively during subsequent perfusion, with the greatest separation between groups occurring during the later sampling time points.

Representative histological assessment provided complementary structural corroboration of the functional phenotype (see Supplementary material online, Figure 12). Haematoxylin and eosin staining demonstrated preservation of the overall myocardial architecture in both groups; however, primary isolated TTS hearts exhibited more prominent interstitial expansion and separation of myocardial fibres compared with control hearts, findings consistent with acute interstitial oedema following prolonged β-adrenergic stimulation. Masson's trichrome staining showed no increase in interstitial collagen, indicating that this interstitial expansion reflected oedema rather than collagen deposition.

Together, these biochemical and histological findings provide independent corroboration of the myocardial involvement underlying the functional phenotype in the isolated-heart TTS model. When integrated with the haemodynamic and echocardiographic data, they indicate preservation of functional and structural integrity compatible with sustained myocardial viability throughout the experimental protocol. Although a modest functional decline is expected during extended crystalloid Langendorff perfusion, control hearts remained comparatively stable across all assessment modalities. In contrast, primary isolated TTS hearts exhibited substantially greater functional deterioration, increased troponin release, and characteristic structural abnormalities, indicating that the observed phenotype exceeded the non-specific, time-dependent changes associated with prolonged ex vivo perfusion.

### Reproducibility

Bootstrapped 95% confidence intervals confirmed reliability of between-group differences (ΔLVDP: −44.2 to −29.1 mmHg; Supplementary material online, Figure 13). Leave-one-rat-out cross-validation demonstrated >90% classification accuracy with stable SHAP rankings across folds, affirming that findings are not driven by individual animals (see Supplementary material online, *Table S3*).

## Discussion

The primary isolated heart TTS model demonstrates that the hallmark features of Takotsubo syndrome, including characteristic apical ballooning, progressive haemodynamic impairment, diastolic dysfunction, and mechanical dyssynchrony, can be induced entirely within an isolated heart, independent of systemic neurohormonal and other extrinsic inputs. The model reproduced apical ballooning and mid-ventricular hypokinesis, with relative preservation or hypercontractility of the basal segments, along with progressive impairment of contractile performance and relaxation. These findings indicate that the myocardium itself contains the substrates necessary for development of a catecholamine-induced TTS-like phenotype when exposed to sufficient adrenergic stress. The model therefore provides a controlled platform for mechanistic investigation and preclinical therapeutic testing in a syndrome for which evidence-based treatment options remain limited.^30^

To our knowledge, this is the first demonstration of the complete functional TTS-like phenotype in a fully isolated heart preparation. The characteristic regional wall-motion pattern, reported in ∼80% of patients with TTS,^1,2,28^ emerged under controlled *ex vivo* conditions and in the absence of circulating hormones, central autonomic input, systemic vascular responses, or extracardiac neurohumoral feedback. This model builds on the *in vivo* isoprenaline protocol pioneered by our group,^20,22^ which has become one of the most widely replicated experimental models of TTS. Unlike *in vivo* models, in which systemic catecholamine surges, vascular responses, and neurohormonal reflexes may obscure the myocardium's direct contribution, the present preparation isolates the cardiac response to catecholaminergic stimulation. Although extracardiac neurohormonal and vascular factors undoubtedly modulate disease severity, clinical presentation, and recovery, the current findings demonstrate that the core regional contractile features of the syndrome can emerge without systemic input.

The haemodynamic evolution of the model was characterized by a reproducible triphasic trajectory. The initial phase was dominated by marked chronotropic and inotropic activation, followed by progressive loss of contractile performance, impaired relaxation, declining myocardial workload, and eventual functional nadir. This temporal sequence resembles the clinical evolution of TTS from an acute stress response through functional instability to maximal dysfunction and subsequent partial recovery. Echocardiography provided complementary confirmation of the phenotype by demonstrating apical ballooning, preserved basal contraction, reduced ejection fraction, less negative global longitudinal strain, increased left ventricular akinesia index, and greater segmental strain heterogeneity. The close agreement between the primary isolated heart TTS model and the secondary isolated heart TTS group, in which the syndrome had first been induced *in vivo* before *ex vivo* perfusion, further supports the reproducibility of the regional functional phenotype.

The present study also highlights important methodological considerations regarding echocardiographic assessment in isolated Langendorff preparations. Because the ventricle contracts under largely unloaded or isovolumic conditions, conventional indices such as ejection fraction cannot be interpreted as direct measures of physiological ventricular pumping capacity. Within this experimental setting, however, EF provides a reproducible, relative functional index that reflects changes in ventricular geometry and regional systolic performance under identical loading conditions. The comparable baseline EF observed across all experimental groups, together with the substantially greater decline in both primary and secondary isolated TTS hearts after catecholamine exposure, supports interpretation of these measurements as relative deterioration of function within the preparation rather than differences in intrinsic baseline performance. The concordant changes in GLS, LVAI, segmental timing, and invasive haemodynamic indices further indicate that the echocardiographic abnormalities reflect genuine impairment of myocardial mechanics within the isolated-heart model.

Biochemical and representative histological assessments strengthened the biological characterization of the model beyond haemodynamic and imaging parameters alone. Serial coronary-effluent measurements demonstrated progressive release of both cTnI and cTnT following β-adrenergic stimulation. Representative H&E and Masson's trichrome sections showed preservation of the overall myocardial architecture, but primary isolated TTS hearts exhibited more prominent interstitial expansion and separation of myocardial fibres, findings compatible with acute interstitial oedema, without evidence of infarction-type replacement fibrosis. These findings indicate that the functional phenotype is accompanied by measurable biochemical and structural myocardial involvement. They should nevertheless be interpreted as qualitative supportive evidence, because formal morphometric analysis, direct measurement of tissue water content, and quantitative assessment of oedema were not performed. When integrated with the haemodynamic and echocardiographic findings, however, the troponin and histological results strengthen confidence that the observed phenotype represents biologically meaningful myocardial dysfunction rather than a purely functional or analytical phenomenon.

The present findings should be interpreted within the biological context of intense β-adrenergic stimulation. Isoprenaline markedly increases heart rate, contractility, calcium cycling, and myocardial energetic demand and may promote calcium overload, oxidative stress, mitochondrial dysfunction, β-adrenergic desensitization, and transient impairment of excitation–contraction coupling. These processes have all been implicated in experimental and clinical studies of catecholamine-mediated myocardial dysfunction and are likely to contribute to the abnormalities observed in the present model. We therefore do not interpret the phenotype as arising from a single isolated mechanism but rather as the integrated consequence of multiple interacting processes triggered by acute catecholamine excess. At the same time, these shared biological responses do not fully explain the differential phenotype observed in the primary isolated heart TTS group. All hearts were studied using the same isolated Langendorff preparation, identical crystalloid perfusion conditions, and comparable experimental handling. Nevertheless, primary isolated TTS hearts consistently developed regional apical dysfunction, progressive haemodynamic impairment, characteristic echocardiographic abnormalities, greater troponin release, and representative structural alterations that exceeded those observed in control hearts. The model should therefore be considered a reproducible catecholamine-induced TTS-like phenotype rather than a complete reproduction of the clinical syndrome.

The temporal evolution of coronary flow also warrants careful interpretation. Coronary flow initially increased following β-adrenergic stimulation, consistent with increased myocardial demand and coronary vasodilation during the early hyperdynamic phase. As the phenotype progressed, coronary flow declined in parallel with worsening haemodynamic performance and echocardiographic abnormalities. Because coronary perfusion was not experimentally manipulated, the present study cannot determine whether reduced coronary flow contributed directly to functional deterioration, developed secondarily to myocardial dysfunction, or interacted bidirectionally with contractile impairment. The corrected directional analyses restricted to directly measured variables did not identify relationships that remained significant after adjustment for multiple comparisons. The coronary-flow findings should therefore be interpreted as part of an integrated temporal association rather than evidence of a direct causal mechanism. Future studies incorporating targeted manipulation of coronary perfusion or vascular resistance will be required to distinguish these possibilities.

Our findings are consistent with, and extend, previous catecholamine-induced rodent models. Paur et al. proposed that a β₂-adrenoceptor signalling shift from Gs to Gi coupling underlies epinephrine-induced apical stunning.^15^ Subsequent work from our group challenged the requirement for this mechanism. Redfors et al. demonstrated that all five tested catecholamines, including norepinephrine and phenylephrine, which lack substantial β₂-adrenoceptor activity, induced TTS-like dysfunction in rats, with the regional pattern determined by afterload rather than stimulation of a specific receptor subtype.^19^ Moreover, contrast echocardiography in a rat model of TTS revealed normal coronary perfusion in the affected regions of the rat heart.^31^ Ali et al. subsequently showed that the phosphodiesterase inhibitor milrinone, which does not act directly on adrenergic receptors, induced TTS-like apical ballooning in the absence of exogenous catecholamines; that selective β₂-blockade with ICI 118551 failed to prevent isoprenaline-induced dysfunction; and that selective β₁-blockade with metoprolol abolished it in a dose-dependent manner.^32^ Taken together, these findings indicate that TTS-like dysfunction is not necessarily contingent on β₂-adrenoceptor/G_i signalling but may reflect a broader response to excessive inotropic and chronotropic stimulation, modulated by ventriculo-arterial coupling. The present study extends this line of evidence by demonstrating that the regional functional phenotype can emerge in an isolated heart, reinforcing the concept that intrinsic myocardial vulnerability to catecholaminergic overstimulation is sufficient to generate the core cardiac features of TTS.

The haemodynamic profile of the model also aligns with clinical observations. TTS induction was followed by impaired contractile performance and pronounced prolongation of relaxation, consistent with invasive findings in human TTS, in which Stiermaier et al. demonstrated systolic dysfunction and prolonged TAU.^6^ Diastolic compliance remained comparatively preserved, suggesting that impaired filling primarily reflected delayed active relaxation rather than fixed structural stiffness. The affected hearts generated less external work despite oxygenated perfusion conditions, consistent with reduced myocardial energetic efficiency and previous reports of metabolic disruption in TTS.^21^ Direct measurements of myocardial oxygen consumption, high-energy phosphate metabolism, mitochondrial function, or substrate utilization were not included in the present study, and future experiments incorporating coronary-effluent metabolomics, oxygen extraction, and tissue bioenergetic analyses will be required to test these hypotheses directly.

The primary isolated heart TTS model provides a controlled platform for systematic pharmacological investigation in a condition for which evidence-based treatment options remain limited.^1,31^ By enabling continuous assessment of drug effects on contractility, relaxation, coronary flow, and regional myocardial mechanics, the model could be used to evaluate both potentially beneficial interventions and agents suspected of aggravating dysfunction, including inotropes^32^ and β-blockers, for which clinical evidence remains conflicting.^33^ Its capacity to manipulate catecholamine type, concentration, timing, loading conditions, and coronary perfusion under standardized conditions creates opportunities for dissecting the physiological basis of clinical heterogeneity in TTS.^9^ The model may therefore complement *in vivo* experiments by allowing controlled interrogation of cardiac-specific mechanisms before candidate interventions are advanced to more complex systems.

Several limitations warrant consideration. The study used healthy young male rat hearts, whereas human TTS occurs predominantly in older postmenopausal women. The exclusive use of male animals is therefore an important limitation and may restrict the ability of the model to reproduce sex-specific influences, including differences in adrenergic receptor expression, calcium handling, and oestrogen-mediated cardioprotection.^15,34^ Rodent myocardium also differs from human myocardium in adrenergic-receptor distribution, excitation–contraction coupling, and calcium kinetics.^35–39^ The isolated-heart preparation lacks central autonomic input, circulating hormones, systemic vascular responses, immune-cell recruitment, and neurohormonal feedback and cannot reproduce the days-long recovery phase observed clinically. The current experiments therefore address the acute intrinsic myocardial response rather than the complete systemic syndrome.

Extended crystalloid Langendorff perfusion is inherently associated with time-dependent deterioration. Albumin was not included in the Krebs–Henseleit perfusate, and prolonged crystalloid perfusion may contribute to interstitial oedema and gradual functional decline. Nevertheless, all groups were exposed to identical perfusion conditions, providing a common experimental background. Control hearts maintained comparatively preserved haemodynamic performance, chamber geometry, limited troponin release, and overall tissue architecture, whereas primary isolated TTS hearts demonstrated substantially greater functional, biochemical, imaging, and representative structural abnormalities. These differences indicate that the observed phenotype exceeded the non-specific effects expected from prolonged perfusion alone, although such effects cannot be eliminated completely.

Additional methodological limitations relate to the derived haemodynamic indices and exploratory analyses. TAU was estimated from dP/dt_min using a predefined surrogate rather than obtained by direct exponential fitting of the ventricular pressure-decay curve. The calculation was applied uniformly across all animals and time points and therefore provides a consistent relative index of relaxation, but it should not be interpreted as a directly measured time constant. Estimated developed tension was calculated using simplified geometric assumptions derived from the Law of Laplace, including constant ventricular radius and wall thickness. These assumptions are unlikely to remain strictly valid during regional dysfunction and apical ballooning; consequently, eDT should be considered an exploratory comparative index rather than a direct quantitative measurement of myocardial wall tension. The multivariate and machine-learning analyses were likewise intended as descriptive, exploratory, and hypothesis-generating tools. PCA, clustering, SHAP, and related analyses describe patterns and relationships among variables within the present dataset but do not establish biological mechanisms or causal pathways.

The biochemical and histological corroboration also has limitations. Histology was intentionally performed as a representative morphological assessment rather than formal quantitative pathological analysis. Myocardial oedema, fibrosis, inflammatory-cell infiltration, vascular injury, oxidative stress, mitochondrial dysfunction, and metabolic alterations were not quantified, and direct wet-to-dry tissue-weight measurements were unavailable. The reproducibility of the functional phenotype across independent experimental series supports the model's robustness; however, the present study remains a proof-of-concept investigation. Validation in female and older animals, quantitative tissue characterization, direct metabolic measurements, and mechanistic intervention studies will be required before broader translational conclusions can be drawn.

In conclusion, the present study demonstrates that the cardinal functional features of Takotsubo syndrome—including regional apical ballooning with relative basal preservation, progressive haemodynamic deterioration, delayed relaxation, and mechanical dyssynchrony,can be reproduced entirely within an isolated heart preparation. These findings establish that intrinsic myocardial responses to acute catecholaminergic stress are sufficient to generate a reproducible

TTS-like phenotype in the absence of systemic neurohumoral input. The primary isolated heart TTS model therefore provides a controlled and complementary experimental platform for mechanistic investigation, physiological characterization, and preclinical therapeutic evaluation, while enabling precise interrogation of cardiac-specific processes that cannot be isolated two readily *in vivo*.

## Lead author biography


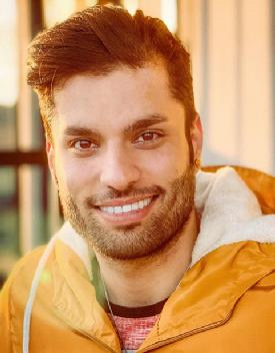


Amirali Nejat, a medical doctor who graduated from Shiraz University of Medical Sciences, Iran, is currently working in translational cardiovascular research at Sahlgrenska Academy, Gothenburg, Sweden.

## Supplementary Material

oeag141_Supplementary_Data

## Data Availability

The data underlying this article are available in the article and its online Supplementary material.
