# Supplementary material for: A primary isolated heart model of Takotsubo syndrome for mechanistic investigation and preclinical therapeutic testing
Source: Eur Heart J Open. 2026 Sep 15;6(5):oeag141. doi: 10.1093/ehjopen/oeag141 (PMC13651902; doi:10.1093/ehjopen/oeag141)
Supplement: Supplementary file 1 [file oeag141_supplementary_data.zip › Figure Legends.docx]

# Figure Legends — Supplement

**Supplementary Figure 1.**

**Distributional profiles of haemodynamic parameters in control and primary isolated TTS hearts.** Kernel density estimation plots showing the pooled distributions of twelve haemodynamic variables recorded over the 360-minute perfusion protocol in control (green, n = 10) and primary isolated heart TTS (red, n = 10). Panels display heart rate, left ventricular systolic pressure, left ventricular end-diastolic pressure, left ventricular developed pressure, maximum and minimum rates of pressure change, isovolumic relaxation constant (TAU), rate-pressure product, coronary flow, estimated coronary vascular resistance, estimated developed tension, and relative rate-pressure product. The broader, frequently multimodal distributions observed in primary isolated heart TTS reflect the interplay of early hyperactivation, transition, and late functional deterioration throughout the full time course. Because repeated measurements from each heart are pooled, these density plots are presented as descriptive distributional summaries and were not used for independent-sample inference.

**Supplementary Figure 2.** **Temporal principal component trajectories of haemodynamic phenotype evolution.** Group-wise mean principal component scores are shown across the 360-minute perfusion protocol for control (green, n = 10) and primary isolated heart TTS (red, n = 10 per). Each connected point represents the mean PCA score for one experimental timepoint to illustrate the direction and temporal progression of the trajectories. PC1 primarily reflects contractile and metabolic/perfusion-related variation, whereas PC2 reflects vascular and diastolic variation. Control hearts remained within a relatively restricted region of PCA space, while primary isolated TTS hearts progressively diverged along a distinct multivariate trajectory during development of the catecholamine-induced TTS-like phenotype. The PCA was exploratory and was used to describe integrated temporal patterning and group separation rather than establish biological mechanisms. Exploratory PERMANOVA with rat-stratified permutations confirmed group separation (p<0.001).

**Supplementary Figure 3.** **SHAP-based ranking of haemodynamic variables contributing to classification of the primary isolated heart TTS phenotype.** Mean absolute SHAP (SHapley Additive exPlanations) values are shown for all haemodynamic variables included in the machine-learning classifier. Larger SHAP values indicate a greater contribution of that variable to discrimination between control and primary isolated TTS hearts. Heart rate (HR) coronary vascular resistance (eCVR) and left ventricular end-diastolic pressure (LVEDP) were the highestranking contributors to model performance, followed by indices of relaxation, systolic function, and myocardial workload. SHAP analysis was performed to facilitate interpretation of the trained classifier and is presented as an exploratory assessment of relative feature importance rather than evidence of causal physiological mechanisms.

**Supplementary Figure 3 Alt Text**: SHAP summary plot of feature importance from XGBoost classification of control vs TTS hearts.

**Supplementary Figure 4. Temporal evolution of SHAP feature importance during development of the primary isolated heart TTS phenotype.** Mean SHAP (SHapley Additive exPlanations) values are shown over the 360-minute perfusion protocol for each haemodynamic variable included in the machine-learning classifier. Higher SHAP values indicate a greater contribution of that variable to discrimination between control and primary isolated TTS hearts at a given time point, whereas a SHAP value of zero indicates no contribution to model discrimination. Background shading denotes the predefined hyperactivation, transition, and functional nadir phases. The trajectories demonstrate that the relative importance of individual haemodynamic variables changes dynamically throughout phenotype development, consistent with the evolving physiological characteristics of catecholamine-induced TTS. These analyses are exploratory and were performed to facilitate interpretation of the trained classifier rather than establish causal physiological mechanisms.

**Supplementary Figure 4 Alt Text**: Temporal changes in SHAP feature contributions during TTS progression.

**Supplementary Figure 5.** **Gaussian mixture model identification of latent haemodynamic states during primary isolated heart TTS progression.** Mean left ventricular developed pressure (LVDP) and diastolic relaxation constant (TAU) are shown across the 360-minute perfusion protocol. Background colours indicate the dominant latent state assigned by the Gaussian mixture model based on the multivariate haemodynamic dataset. The unsupervised model identified three sequential temporal states that closely corresponded to the hyperactivation, transition, and functional nadir phases defined from the physiological analyses. The progressive decline in LVDP together with increasing TAU illustrates the transition from preserved systolic function and rapid relaxation to progressive contractile failure and impaired diastolic relaxation during development of the isolated heart TTS phenotype.

**Supplementary Figure 5 Alt Text**: Gaussian Mixture Model clustering of physiological states in primary isolated heart TTS for LVDP and TAU.

**Supplementary Figure 6. Temporal evolution of the complete haemodynamic phenotype across Gaussian mixture model-derived latent states.** Mean trajectories of all haemodynamic variables are shown during the 360-minute perfusion protocol for the primary isolated heart TTS group. Background colours correspond to the latent states identified by the Gaussian mixture model (Supplementary Figure 6), which closely aligned with the hyperactivation, transition, and functional nadir phases. The coordinated changes observed across chronotropic, systolic, diastolic, perfusion-related, and derived haemodynamic variables illustrate the progressive evolution of the isolated heart TTS phenotype throughout the experiment. The latent states represent statistical clusters identified by the unsupervised Gaussian mixture model and are interpreted in the context of the physiological analyses presented in the main manuscript.

**Supplementary Figure 6 Alt Text:** Multi-parameter Gaussian Mixture Model cluster assignments across all haemodynamic variables.

**Supplementary Figure 7: Unsupervised hierarchical clustering of hearts and correlation structure of haemodynamic parameters.** **(A)** Hierarchical clustering dendrogram of individual control and primary isolated TTS hearts based on their standardized multivariate haemodynamic profiles. Leaf labels indicate individual rat identifiers. The analysis separated control and primary isolated heart TTS preparations into two principal branches, demonstrating distinct integrated haemodynamic profiles between groups. The horizontal bars below the dendrogram identify control and primary isolated TTS hearts. **(B)** Hierarchically clustered Pearson correlation matrix of the 12 haemodynamic variables included in the analysis. Correlations were calculated from rat-level summary profiles rather than pooled repeated timepoints. Cell values indicate Pearson correlation coefficients; red denotes positive correlation and blue denotes negative correlation. The clustered matrix identified a predominantly contractile/workload-related grouping and a predominantly diastolic/vascular grouping with inverse relationships between them. The correlation structure is presented as an exploratory description of coordinated parameter behaviour and does not establish biological regulation or causation. Several displayed indices are mathematically derived from other variables, and correlations involving those indices should therefore not be interpreted as independent physiological associations.

**Supplementary Figure 7 Alt Text**: Hierarchical clustering dendrogram and correlation heatmap of 12 physiological parameters in TTS hearts.

**Supplementary Figure 8. Segmental heterogeneity of myocardial contraction timing in isolated heart preparations.** (A) Standard deviation of longitudinal time-to-peak (TPK) strain across six ventricular segments in control, primary isolated heart TTS, and secondary isolated heart TTS hearts. (B) Standard deviation of radial TPK strain across the same ventricular segments. Higher standard deviation values indicate greater temporal dispersion of regional contraction and increased mechanical dyssynchrony. Both primary and secondary isolated heart TTS groups demonstrated substantially greater regional variability than controls, consistent with impaired synchrony of ventricular contraction. Values are presented descriptively to illustrate regional heterogeneity.

**Supplementary Figure 8 Alt Text**: Segmental strain heterogeneity (time-to-peak strain dispersion) in control and both TTS groups.

**Supplementary Figure 9. Correlations between echocardiographic and haemodynamic indices.** Pearson correlation coefficients between invasive haemodynamic variables and the principal echocardiographic measures of ventricular function (ejection fraction, global longitudinal strain, and left ventricular akinesia index). Red indicates positive correlations and blue indicates negative correlations, with colour intensity proportional to the magnitude of the correlation coefficient. Reduced systolic function and increased regional wall-motion abnormalities were associated with progressive deterioration of invasive haemodynamic indices, supporting concordance between echocardiographic and haemodynamic assessment in the isolated-heart model.

Correlations describe associations and should not be interpreted as evidence of causality.

**Supplementary Figure 9 Alt Text**: Correlation matrix heatmap between echocardiographic and haemodynamic parameters.

**Supplementary Figure 10. Integrated principal component analysis (PCA) of haemodynamic and echocardiographic variables.** Principal component analysis was performed using standardized invasive haemodynamic variables (HR, LVSP, LVEDP, LVDP, dP/dt_max, dP/dt_min, RPP, coronary flow, eCVR, TAU, eDT and rRPP) together with echocardiographic indices (EF, global longitudinal strain [GLS] and left ventricular akinesia index [LVAI]). Individual points represent individual hearts, coloured according to experimental group (control, green; primary isolated heart TTS, red). Arrows indicate variable loadings, with arrow direction representing the contribution of each variable to the principal components and arrow length indicating the relative strength of that contribution. PC1 explained **77.4%** of the total variance and primarily reflected the continuum from preserved contractile and perfusion function (RPP, LVSP, LVDP, coronary flow and eDT) to progressive ventricular dysfunction. PC2 explained **11.8%** of the variance and was predominantly associated with diastolic and echocardiographic features, including LVAI, GLS and relaxation-related indices. The PCA demonstrates clear multivariate separation between control and primary isolated TTS hearts, indicating that invasive haemodynamic and echocardiographic measurements provide concordant characterization of the isolated-heart TTS phenotype.

**Supplementary Figure 10 Alt Text**: Combined PCA biplot of haemodynamic and echocardiographic variables showing group separation.

**Supplementary Figure 11**. Serial coronary effluent concentrations of cardiac troponin I (cTnI; A) and cardiac troponin T (cTnT; B) measured in control (green, n = 10) and primary isolated heart

TTS (red, n = 10) during the early phase of the perfusion protocol. Data are presented as mean ± SEM (n = 3 per group). The shaded region indicates the 15-minute isoprenaline infusion period.

**Supplementary Figure 11 Alt Text:** Early biochemical corroboration of myocardial injury in the isolated-heart TTS model.

**Supplementary Figure 12**. Representative whole-heart sections stained with haematoxylin and eosin (H&E) and Masson's trichrome are shown for control hearts (A–B) and primary isolated TTS hearts (C–D). H&E staining demonstrates the overall myocardial architecture, whereas Masson's trichrome was used to assess structural myocardial morphology and collagen distribution. Scale bars are shown in each panel. Representative images are presented as supportive structural corroboration of the functional phenotype.

**Supplementary Figure 12 Alt Text:** Representative histological assessment of control and primary isolated TTS hearts following prolonged Langendorff perfusion.

**Supplementary Figure 13**. Bootstrapped 95% confidence intervals for changes (Δ) in all parameters from baseline to 360 minutes in control versus primary isolated heart TTS groups (n=10/group), demonstrating reliable trends in TTS dysfunction. Non-overlapping intervals confirm reliability of between-group differences.

**Supplementary table 1**: **Echocardiographic comparison between control, secondary isolated heart TTS, and primary isolated heart TTS groups.**

Summary of echocardiographic parameters including ejection fraction (EF%), global longitudinal strain (GLS%), and left ventricular akinesia index (LVAI%) across control, secondary isolated heart TTS, and primary isolated heart TTS groups. Statistical comparisons were performed using one-way ANOVA followed by Tukey’s post hoc testing for multiple comparisons. Both TTS groups demonstrated significantly impaired systolic function and increased regional akinesia compared with controls, while no significant differences were observed between secondary isolated heart TTS and primary isolated heart TTS groups, associated with phenotypic concordance between models. GLS values are conventionally expressed as negative values; less negative GLS indicates impaired systolic function. Data are presented as mean ± SEM.

**Supplementary table 2: Principal component loading matrix and explained variance for integrated haemodynamic and echocardiographic PCA.**

Principal component analysis (PCA) integrating endpoint haemodynamic and echocardiographic variables in control and primary isolated heart TTS. The table presents principal component loading coefficients, eigenvalues, explained variance, and cumulative variance for PC1 and PC2. PC1 primarily reflected contractile and perfusion-related parameters, whereas PC2 was associated with diastolic and strain-derived features. To reduce redundancy and avoid multicollinearity within the integrated PCA model, ejection fraction (EF%) was excluded because of strong covariance with GLS and LVAI. PCA was performed on standardized variables using endpoint data at 360 minutes. Because GLS is conventionally expressed as a negative value, positive PCA loadings correspond to less negative (more impaired) strain values.

**Supplementary Table 3. Leave-one-rat-out sensitivity analysis of haemodynamic group differences.**

Sequential leave-one-rat-out (LORO) sensitivity analysis demonstrating robustness of haemodynamic group differences following exclusion of individual animals. For each iteration, one rat was removed and the mean difference between primary isolated heart TTS and control groups was recalculated across all recorded timepoints and physiological parameters. Consistent directional changes and low variability across folds confirmed that the observed phenotype was not driven by individual animals, identifying the reproducibility and stability of the model.

1

2 **Supplementary Figure 13 Alt Text**: Bootstrapped 95% confidence intervals for haemodynamic
3 changes from baseline to 360 minutes.
