# Supplementary material for: A primary isolated heart model of Takotsubo syndrome for mechanistic investigation and preclinical therapeutic testing
Source: Eur Heart J Open. 2026 Sep 15;6(5):oeag141. doi: 10.1093/ehjopen/oeag141 (PMC13651902; doi:10.1093/ehjopen/oeag141)
Supplement: Supplementary file 1 [file oeag141_supplementary_data.zip › Supplementary_Figure_Histology_HE_MT.pdf]

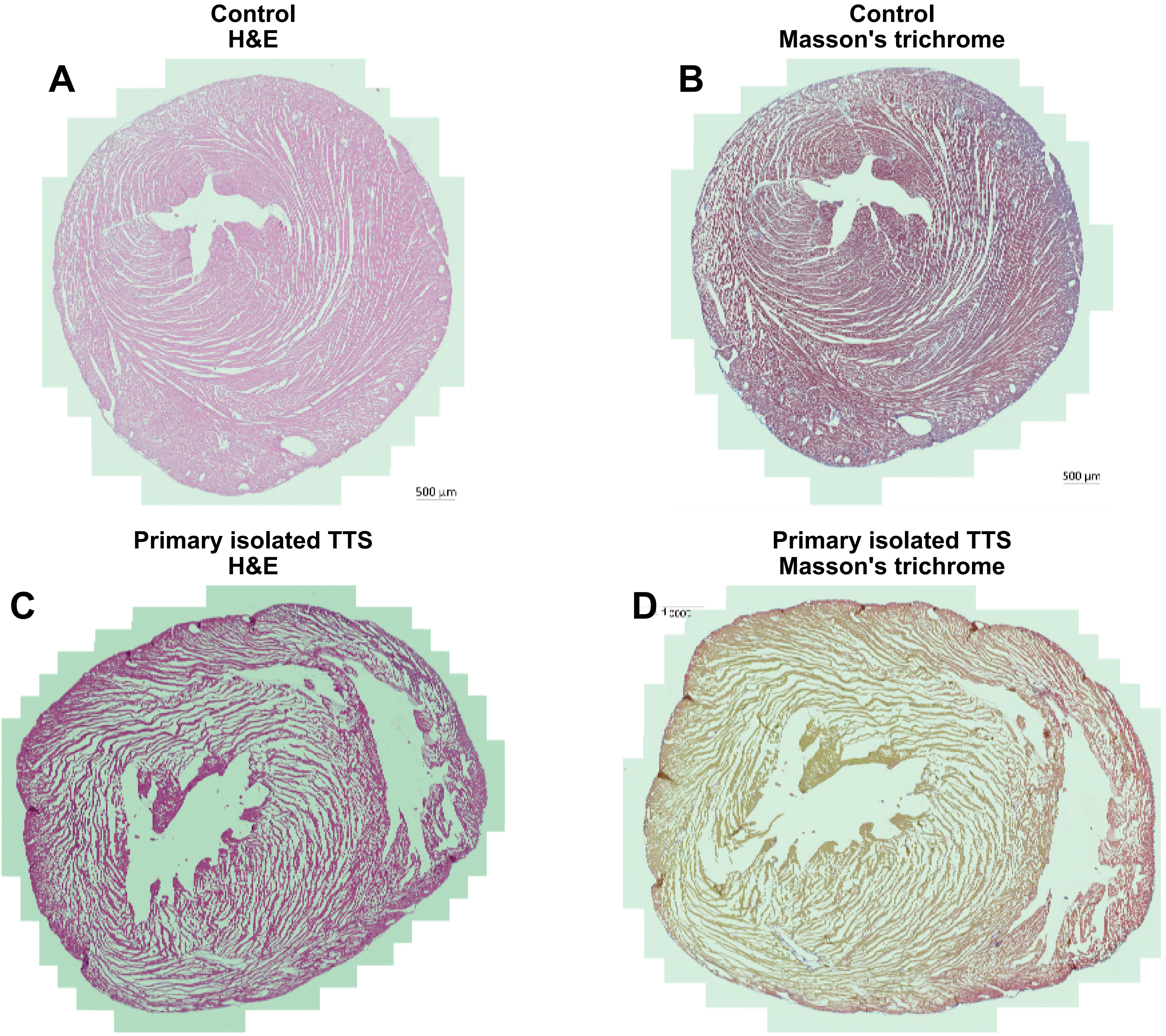
Control
H&E
A
500 µm
Control
Masson's trichrome
B
500 µm
Primary isolated TTS
H&E
C
Primary isolated TTS
Masson's trichrome
D
